# First in human measurements of abscess cavity optical properties and methylene blue uptake prior to photodynamic therapy by *in vivo* diffuse reflectance spectroscopy

**DOI:** 10.1117/1.JBO.29.2.027002

**Published:** 2024-02-27

**Authors:** Md Nafiz Hannan, Ashwani K. Sharma, Timothy M. Baran

**Affiliations:** a University of Rochester, Department of Physics and Astronomy, Rochester, New York, United States; b University of Rochester Medical Center, Department of Imaging Sciences, Rochester, New York, United States; c University of Rochester, Department of Biomedical Engineering, Rochester, New York, United States; d University of Rochester, The Institute of Optics, Rochester, New York, United States

**Keywords:** diffuse reflectance spectroscopy, intra-abdominal abscess, methylene blue, photodynamic therapy, optical properties, hemodynamics

## Abstract

**Significance:**

Efficacious photodynamic therapy (PDT) of abscess cavities requires personalized treatment planning. This relies on knowledge of abscess wall optical properties, which we report for the first time in human subjects.

**Aim:**

The objective was to extract optical properties and photosensitizer concentration from spatially resolved diffuse reflectance measurements of abscess cavities prior to methylene blue (MB) PDT, as part of a phase 1 clinical trial.

**Approach:**

Diffuse reflectance spectra were collected at the abscess wall of 13 human subjects using a custom fiber-optic probe and optical spectroscopy system, before and after MB administration. A Monte Carlo lookup table was used to extract optical properties.

**Results:**

Pre-MB abscess wall absorption coefficients at 665 nm were $0.15\pm 0.1\;{\mathrm{cm}}^{-1}$ (0.03 to $0.36\;{\mathrm{cm}}^{-1}$) and $10.74\pm 15.81\;{\mathrm{cm}}^{-1}$ (0.08 to $49.3\;{\mathrm{cm}}^{-1}$) post-MB. Reduced scattering coefficients at 665 nm were $8.45\pm 2.37\;{\mathrm{cm}}^{-1}$ (4.8 to $13.2\;{\mathrm{cm}}^{-1}$) and $5.6\pm 2.26\;{\mathrm{cm}}^{-1}$ (1.6 to $9.9\;{\mathrm{cm}}^{-1}$) for pre-MB and post-MB, respectively. Oxygen saturations were found to be 58.83%±35.78% (5.6% to 100%) pre-MB and 36.29%±25.1% (0.0001% to 76.4%) post-MB. Determined MB concentrations were 71.83±108.22  μM (0 to 311  μM).

**Conclusions:**

We observed substantial inter-subject variation in both native wall optical properties and MB uptake. This underscores the importance of making these measurements for patient-specific treatment planning.

## 1 Introduction

Abscesses, which consist of a purulent collection surrounded by a fibrous pseudo-capsule, form as a result of host immune and inflammatory response to an acute bacterial infection.^1^ These localized pockets of infection can include planktonic microorganisms in the purulent fluid, as well as biofilm populations growing on the abscess wall. Without treatment, abscess cavities can rupture or spread systemically, leading to high rates of morbidity and mortality.^2^ Although image-guided percutaneous drainage has become the standard of care for abscesses that do not respond to antibiotics alone,^3^ abscess resolution rates can be low in certain cases^4^ and complications following drainage are a persistent problem.^5^^,^^6^ Even in the case of technically successful drainage, bacteria can remain in the cavity in undrained fluid or as biofilms growing on the abscess wall. The current standard of care therefore relies on antibiotics to eliminate this residual bacterial load. However, the occurrence rate of antibiotic-resistant bacteria is on the rise, with multi-drug resistant pathogens increasingly found in abscess aspirates.^7^^,^^8^ Alternative treatments are therefore needed.

Photodynamic therapy (PDT), which produces an antimicrobial effect through the photochemical production of reactive oxygen species, may be a powerful adjunct to drainage in the treatment of infected abscesses. Multiple studies have shown that PDT is efficacious *in vitro* against bacterial species that are typically found in abscess cavities.^7^^,^^9^ Based on these promising results, we completed a phase 1 clinical trial studying the safety and feasibility of PDT with the photosensitizer methylene blue (MB) at the time of abscess drainage (ClinicalTrials.gov Identifier: NCT02240498). The primary clinical outcomes of this trial are reported elsewhere.^8^ Here, we focus on the results of optical spectroscopy measurements made immediately prior to PDT.

PDT efficacy is largely determined by the combination of the absorbed light dose and the photosensitizer concentration.^10^ The absorbed light dose is determined by the treatment laser power and the optical properties, absorption and scattering, of the target tissue. If the tissue optical properties are known, forward modeling can be used to determine the distribution of light dose within the target tissue. Given a target light dose, patient-specific treatment plans can then be generated to ensure that the entire target region receives an efficacious light dose.

This type of patient-specific PDT treatment planning has been widely applied for interstitial PDT of cancer.^11^ For example, Davidson et al. used the treatment diffuser fibers to extract optical properties of the prostate based on a diffusion model.^12^ These optical properties were then used to design treatment plans that delivered a threshold dose to the prostate plus margin. Lietke et al. used a similar approach in designing treatment plans for malignant glioma recurrences.^13^ In the context of a larger body space, Dupre et al. reported on the performance of surface contact spatially-resolved diffuse reflectance spectroscopy prior to PDT of the pleural cavity.^14^ These measurements, along with real-time fluence monitoring,^15^ were used to deliver uniform light doses to highly heterogeneous subjects.

While other groups have reported optical property measurements within the human abdomen,^16^^,^^17^ the optical properties of human abscesses have never been measured, making accurate light dose modeling impossible. To remedy this, we have designed, built, and validated a spatially resolved diffuse reflectance spectroscopy system incorporating multiple optical fibers within a 2 mm outside diameter package.^18^^,^^19^ As described above, many prior applications typically rely on multiple distinct spectroscopy fibers,^12^^,^^13^^,^^20^ large area of access to the treatment site,^14^^,^^15^ or bulky, rigid fiber optic probes^21^^–^^23^ in order to extract patient-specific optical properties for treatment planning. In the case of abscesses, access is generally provided by a small drainage catheter and avoidance of abscess rupture is paramount. The small outside diameter (∼2  mm) and flexibility of the fiber-optic probe are therefore crucial for integration into the clinical workflow.

In this study, we performed diffuse reflectance spectroscopy immediately prior to PDT on a series of 13 subjects treated as part of the parent Phase 1 clinical trial. These spectroscopy measurements were taken both before and after MB administration, to quantify native abscess wall optical properties and MB uptake. This represents the first report on the optical properties of human abscess cavities, as well as the retention of MB following a 10 min incubation. These optical property measurements set the stage for patient-specific treatment planning,^24^ building on our retrospective study results.^25^^,^^26^ We previously reported preliminary results for the first three subjects measured in this study.^27^ Here, we present the full results for the entire study cohort, including additional analyses.

## 2 Materials and Methods

### 2.1 Participants and Regulatory Approval

Spectroscopy data were collected from subjects enrolled in a phase 1 clinical trial examining the safety and feasibility of MB PDT performed at the time of percutaneous abscess drainage (ClinicalTrials.gov Identifier: NCT02240498). The primary results of this clinical trial are reported separately.^8^ Of the 18 subjects treated in this trial, optical spectroscopy data were collected from the final 13 subjects as described below. For the first five subjects treated, funding was not available to construct the spectroscopy system described in Sec. 2.2 and perform spectroscopy measurements. All study procedures were approved by the Research Subjects Review Board at the University of Rochester Medical Center, and written informed consent was provided by all subjects.

### 2.2 Diffuse Reflectance Spectroscopy System

**Fig. 1:**
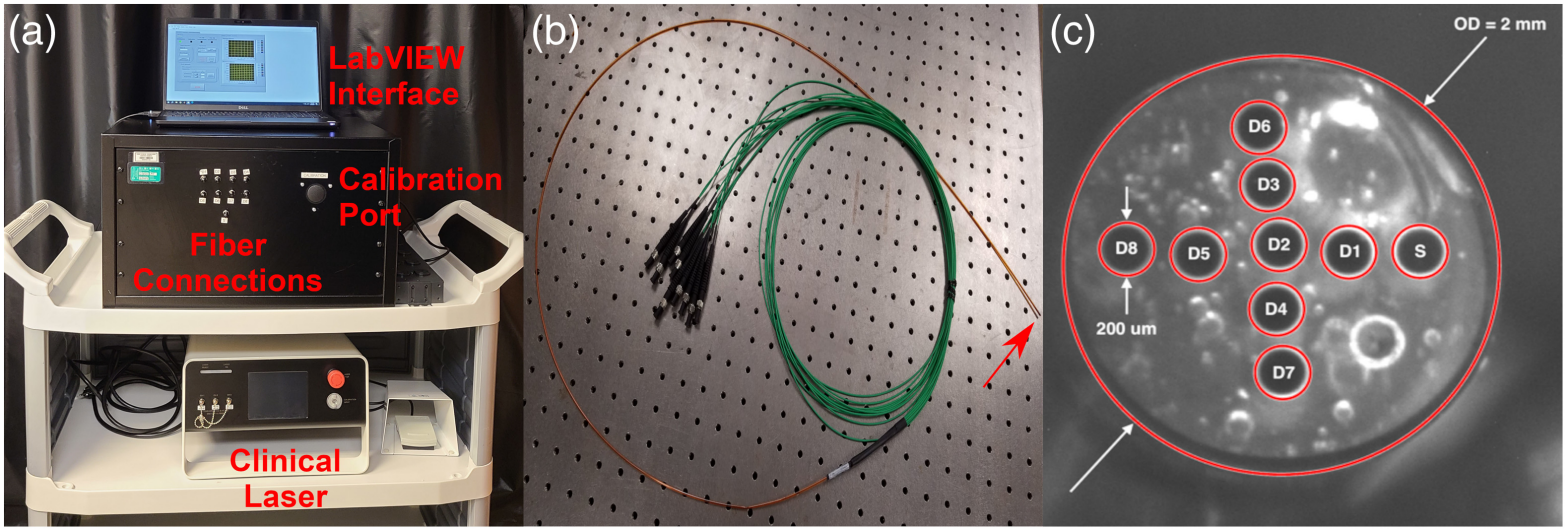
(a) Image of spectroscopy system showing fiber connections for source and detector fibers, port for integrating sphere calibration, LabVIEW interface, and the laser used for clinical PDT. (b) Image of fiber optic probe, with distal tip indicated by red arrow. (c) Image of fiber optic probe distal face showing the 200  μm diameter fibers used for delivery and detection of light. The source fiber is labeled “S,” detector fibers are labeled “D,” and outside diameter is abbreviated as “OD.”

Spatially resolved diffuse reflectance data at the abscess wall were collected using an optical spectroscopy system that was described in detail previously,^18^ as shown in Fig. 1(a). Briefly, this system consists of an optical switch (FSM 1x2, Piezosystem Jena, Inc., Hopedale, Massachusetts, United States) that routes either broadband white light (HL-2000-HP-FHSA, Ocean Optics, Inc., Largo, Florida, United States) or laser light at 640 nm (OBIS FP 1193841, Coherent, Inc., Santa Clara, California, United States) to the source fiber on the optical probe displayed in Figs. 1(b) and 1(c). Light transmitted by each of the eight detector fibers is sequentially routed to a spectrometer (QE Pro, Ocean Optics) for detection via another optical switch (FSM 1x8, Piezosystem Jena, Inc.). Detected spectra were corrected for dark background and integration time, and were each divided by a corresponding calibration measurement made using an integrating sphere (3P-GPS-020-SF, Labsphere, Inc., North Sutton, NH) incorporated into the system housing. Details of the calibration procedure are reported elsewhere.^18^ While we have examined the fluorescence data collected by this system pre-clinically,^19^ the focus of the current study is the analysis of diffuse reflectance data.

The fiber optic probe used for delivery of incident light and detection of diffuse reflectance (Pioneer Optics Company, Bloomfield, Connecticut, United States) is shown in Fig. 1(b). This flexible probe has an outside diameter of 2 mm, to allow it to be inserted through the standard of care drainage catheter used for percutaneous abscess drainage, as described in Sec. 2.3. To determine the source–detector separations necessary for determination of optical properties from measured spectra, images were captured of the probe face using a stereomicroscope with a large working distance (SMZ1500, Nikon Instruments, Inc., Melville, New York, United States). These images were captured with and without a United States Air Force target (USAF 1951 1X, Edmund Optics, Inc., Barrington, New Jersey, United States) in the frame to translate pixel coordinates to distance. These source-detector separations ranged from 320 to 1310  μm, as shown in Fig. 1(c).

As described by Bridger et al., these source-detector separations were used to generate a Monte Carlo lookup table for extraction of optical properties from measured spectra.^18^ This pre-clinical validation demonstrated root mean square errors of 0.26 to 0.52  μM for MB concentration recovery and 0.86 to $2.7\;{\mathrm{cm}}^{-1}$ for reduced scattering coefficient ($\mu_{s}^{\prime }$) recovery. This prior publication used a lower resolution color camera to determine fiber positions, whereas the current results utilize the more precise positions determined by microscopic evaluation. Otherwise, the procedure was identical to the previously reported multispectral fitting method. Based on visual inspection of measured spectra, absorption spectra were fit as a superposition of oxy- and deoxy-hemoglobin, MB monomer, and MB dimer absorption spectra. MB dimer absorption was included as MB dimerizes at high concentrations and there is an affiliated change in the absorption spectrum.^28^ Reduced scattering spectra were assumed to follow a power-law relationship of the form $\mu_{s}^{\prime }=a{\left(\lambda /\lambda_{0}\right)}^{-b}$, where λ corresponds to wavelength in nm and $\lambda_{0}$ is a normalization wavelength set to 665 nm. Quality of fitting is reported as the sum of root squared error (SRSE) across detector fibers between measured and simulated reflectance spectra.

### 2.3 Clinical Data Collection

All subjects first had their abscess drained using standard of care image-guided percutaneous drainage, as described in the main trial report.^8^ Aspirated fluid was processed by the Clinical Microbiology Laboratory, including identification of isolates and determination of antibiotic susceptibility. Growth of isolates was scored on a semi-quantitative scale ranging from 0 (no growth) to 4+ (heavy growth). The standard of care procedure utilized CT guidance for 16 subjects and ultrasound guidance for 2 subjects. Immediately following drainage, the cavity was first flushed twice with sterile saline. The disinfected optical probe was then removed from the bag and the proximal fiber ends connected to the spectroscopy system. The distal end was then held in place at the entrance port of the system integrating sphere by the interventional radiologist. Calibration spectra were captured at each detector fiber for both broadband and laser illumination, with an integration time of 100 ms for broadband illumination and 50 ms for laser illumination. These calibration spectra were used to correct patient data as described in Sec. 2.2.

In order to measure patient optical properties, the interventional radiologist advanced the optical probe through the standard of care drainage catheter until it made gentle contact with the abscess wall. Surface contact was insured through tactile feedback by the study doctor, and by monitoring the signal detected at the closest detector fiber in real time. As described by Chen et al.,^29^ detected reflectance increases as the probe approaches the surface before rapidly decreasing when surface contact is made. This is due to the transition from specular to diffuse reflectance.

Once surface contact was made, diffuse reflectance and fluorescence spectra were captured at each detector fiber sequentially. For each fiber, an initial integration time of 50 ms was used. Assuming a linear relationship between integration time and detected signal, the maximum magnitude of the detected signal was used to calculate the integration time that would result in 50% of the dynamic range of the spectrometer being utilized. This was chosen to compromise between signal to noise ratio and measurement time. A measurement was then performed at this calculated integration time. Dark spectra were also collected at this point as described in Sec. 2.2 using the same integration times by disabling the source switch. If the captured spectra were not of sufficient quality, the probe was withdrawn and replaced by the study doctor, and spectroscopy was repeated. This measurement was meant to capture native abscess wall optical properties prior to MB infusion and is referred to as the “pre-MB” measurement elsewhere.

Following this, 0.1% MB (BPI Labc, LLC, Largo, Florida, United States) was infused into the abscess cavity and incubated for 10 min to allow for bacterial uptake. A 10 min incubation interval was selected based on prior reports of *in vitro* PDT efficacy against multiple bacterial isolates from human abscess aspirates using this incubation time.^7^ This short interval was also selected to improve specificity of MB uptake, based on the rapid internalization of cationic photosensitizers by bacteria relative to host cells.^30^ MB was then aspirated and the cavity was flushed twice with sterile saline for all subjects. The optical probe was then re-inserted through the drainage catheter into gentle contact with the abscess wall, and measurements were repeated as described above. This measurement was meant to capture the uptake of MB and is referred to as the “post-MB” measurement throughout.

After the completion of optical spectroscopy measurements, the optical probe was removed and PDT was performed as described in the main clinical trial report.^8^ This consisted of an infusion of a lipid emulsion to scatter treatment light through the irregular abscess cavity, insertion of a sterile optical fiber, and delivery of laser light at 665 nm.

### 2.4 Data Processing

As described above, collected spectra were corrected for dark background and integration time, and divided by corresponding spectra collected at the entrance port of the system integrating sphere. Optical property spectra were extracted using the Monte Carlo lookup table approach described in Sec. 2.2. Whereas pre-clinical validation of the system used all eight detector fibers, high-quality data could not be collected at all detector fibers for all subjects in clinical measurements. This was mainly due to poor surface contact between specific detector fibers and the abscess wall, even after repositioning of the optical probe. Additionally, for subjects with high MB uptake, spectra collected at larger source-detector separations did not have sufficient signal to noise ratio for analysis due to major attenuation by MB absorption. In cases where spectra at individual detectors were not usable, these detected fibers were excluded from the optical property inversion. As we have shown previously, utilization of fewer source-detector separations does not significantly impact the accuracy of optical property recovery, as long as data from at least 4-5 detector fibers are included.^31^^,^^32^ This was achievable for all but the first subject, resulting in 13 pre-MB measurements and 12 matched post-MB measurements.

### 2.5 Statistical Analysis

Continuous values are summarized across subjects as mean ± standard deviation, whereas categorical values are summarized as proportions with 95% confidence intervals. Demographic information was compared between the 13 subjects that received optical spectroscopy and the five treated prior to construction of the optical spectroscopy system using the Mann–Whitney test for continuous variables and Fisher’s exact test for categorical values. Extracted optical properties at 665 nm and ${\mathrm{SO}}_{2}$ were compared between pre- and post-MB measurements using the Wilcoxon signed-rank test. Correlation between MB concentration and change in ${\mathrm{SO}}_{2}$ was calculated using the Spearman correlation coefficient. Optical properties were compared between abscess locations using the Kruskal–Wallis test, with Dunn’s test for pairwise comparisons. The Mann–Whitney test was used to compare MB concentration between those with heavy bacterial growth reported for their standard of care abscess aspirate (4+) to those with less growth (<4). A p value less than 0.05 was considered significant, and all analyses were performed in GraphPad PRISM (v6, GraphPad Software, Inc., San Diego, California, United States) and MATLAB (R2022b, The Mathworks, Inc., Natick, Massachusetts, United States).

## 3 Results

### 3.1 Subject Demographics

Subject demographics for all subjects that received PDT, as well as separated by whether optical spectroscopy measurements were made, are included in Table 1. As can be seen, there were no significant differences in demographics between subjects that did and did not receive optical spectroscopy. For the 13 subjects described in this report, 38.5% were male and the average age was 56.9±20.3 years.

**Table 1:** Subject demographics for overall sample and separated by whether spectroscopy was performed.

	Overall sample (n=18)	Optical spectroscopy performed (n=13)	Optical spectroscopy not performed (n=5)	P value
Age	60.1 ± 18.3	56.9 ± 20.3	68.6 ± 7.6	0.20
Sex (% male)	44.4% (21.5% to 69.2%)	38.5% (13.9% to 68.4%)	60.0% (14.7% to 94.7%)	0.61
BMI	29.3 ± 5.9	30.0 ± 6.3	25.4 ± 6.1	0.39
Abscess volume (mL)	36.8 ± 40.6	25.2 ± 22.0	67.0 ± 63.0	0.18

P values are the results of the Mann–Whitney test for continuous variables and Fisher’s exact test for categorical variables, comparing those for which optical spectroscopy was and was not performed.

### 3.2 Representative Data from Two Subjects

**Fig. 2:**
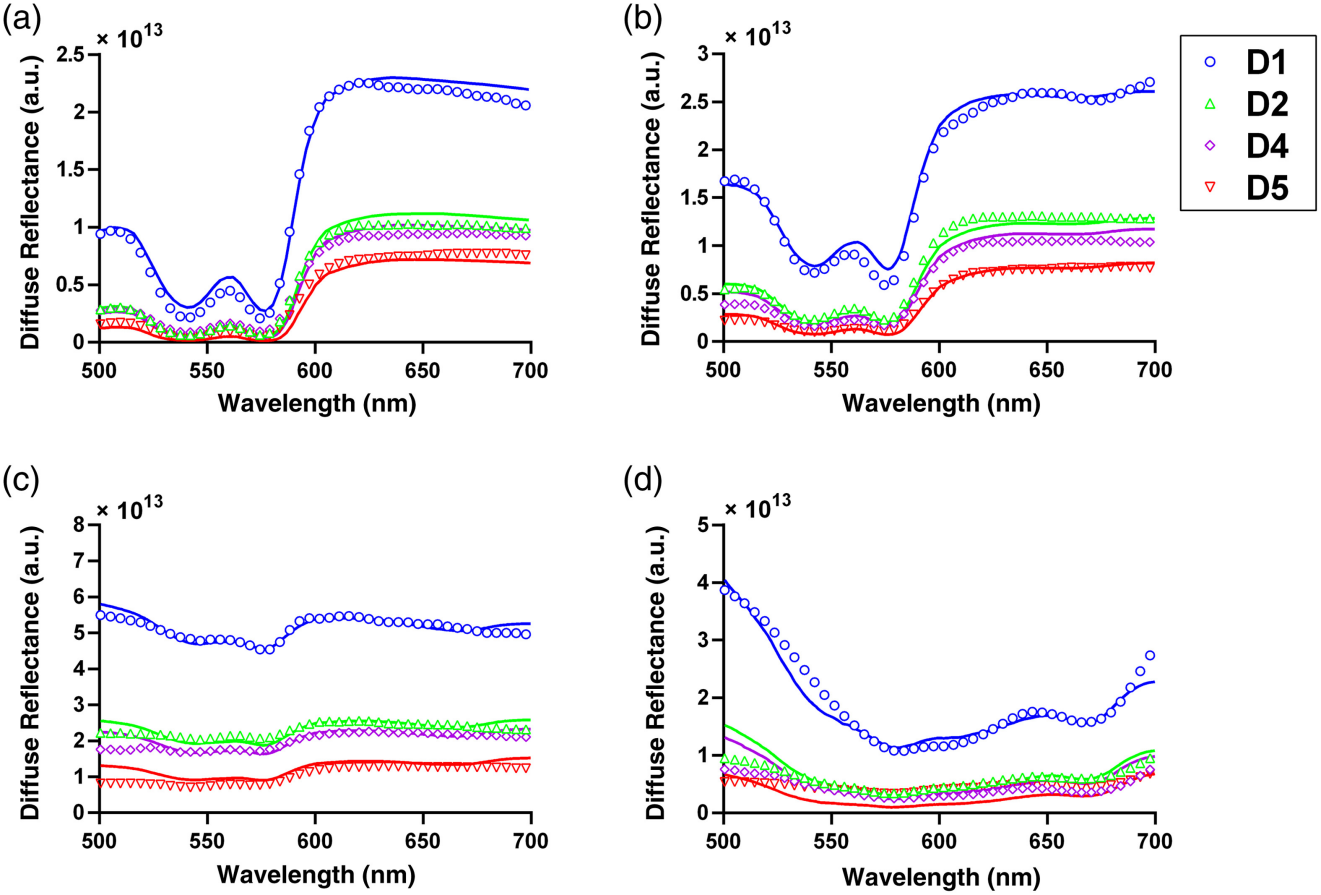
Corrected diffuse reflectance data (open circles) and fits (solid line) for detector fibers 1, 2, 4, and 5 for subject 3 (a) pre-MB $\left(\mathrm{SRSE}=1.18\times 10^{15}\right)$ and (b) post-MB $\left(\mathrm{SRSE}=1.13\times 10^{15}\right)$, and subject 8 (c) pre-MB ($\mathrm{SRSE}=1.32\times 10^{15})$ and (d) post-MB ($\mathrm{SRSE}=1.36\times 10^{15}$). Colors and symbols indicate different detector fibers.

Representative diffuse reflectance spectra for two subjects are shown in Fig. 2, with fully corrected data shown as open circles and best fit spectra shown as solid lines. As can be seen in Figs. 2(a) and 2(c), pre-MB data were accurately fit with a super-position of oxy- and deoxy-hemoglobin absorption and power-law reduced scattering. Post-MB data, as shown in Figs. 2(b) and 2(d), were well fit by the addition of MB monomer and dimer spectra.

**Fig. 3:**
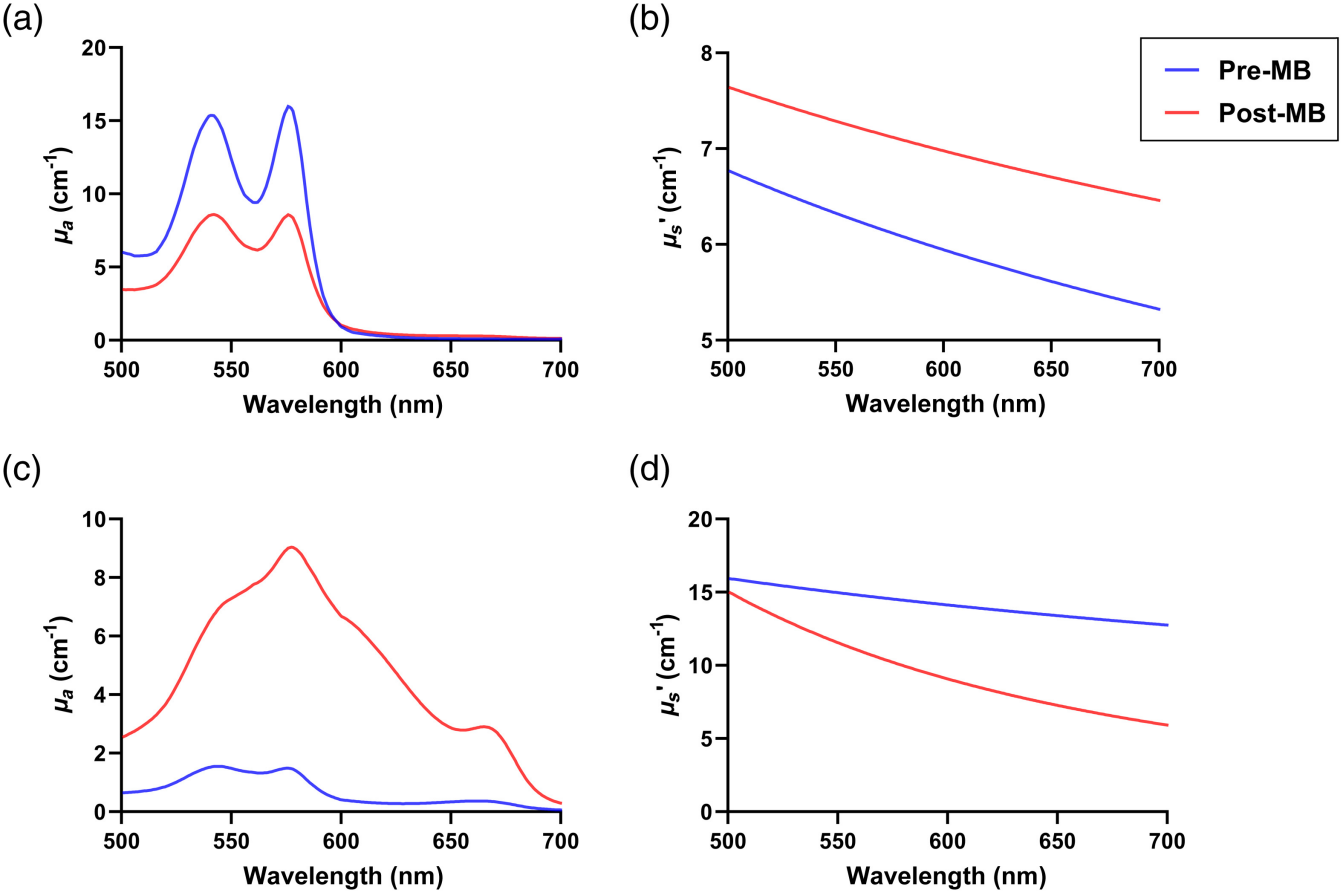
Recovered (a) absorption ($\mu_{a}$) and (b) reduced scattering ($\mu_{s}^{\prime }$) spectra for subject 3 shown in Figs. 2(a) and 2(b). Recovered (c) $\mu_{a}$ and (d) $\mu_{s}^{\prime }$ spectra for subject 8 shown in Figs. 2(c) and 2(d). Blue and red lines represent pre-MB and post-MB conditions, respectively.

These fits were used to extract the optical property spectra shown in Fig. 3. As can be seen, there was variability between subjects in both pre-MB optical properties and MB uptake. Figures 3(a) and 3(c) demonstrate large differences in absorption spectra between subjects for both pre-MB and post-MB conditions. In both subjects, oxy- and deoxy-hemoglobin were the main absorbers.

In the post-MB condition for subject 3, oxy- and deoxy-hemoglobin remained the dominant absorbers. For subject 8, MB monomer and dimer dominated the post-MB absorption spectra, with some contributions from oxy- and deoxy- hemoglobin. Figures 3(b) and 3(d) illustrate notable differences in reduced scattering spectra between subjects for pre-MB and post-MB conditions.

### 3.3 Native Abscess Wall Optical Properties

**Fig. 4:**
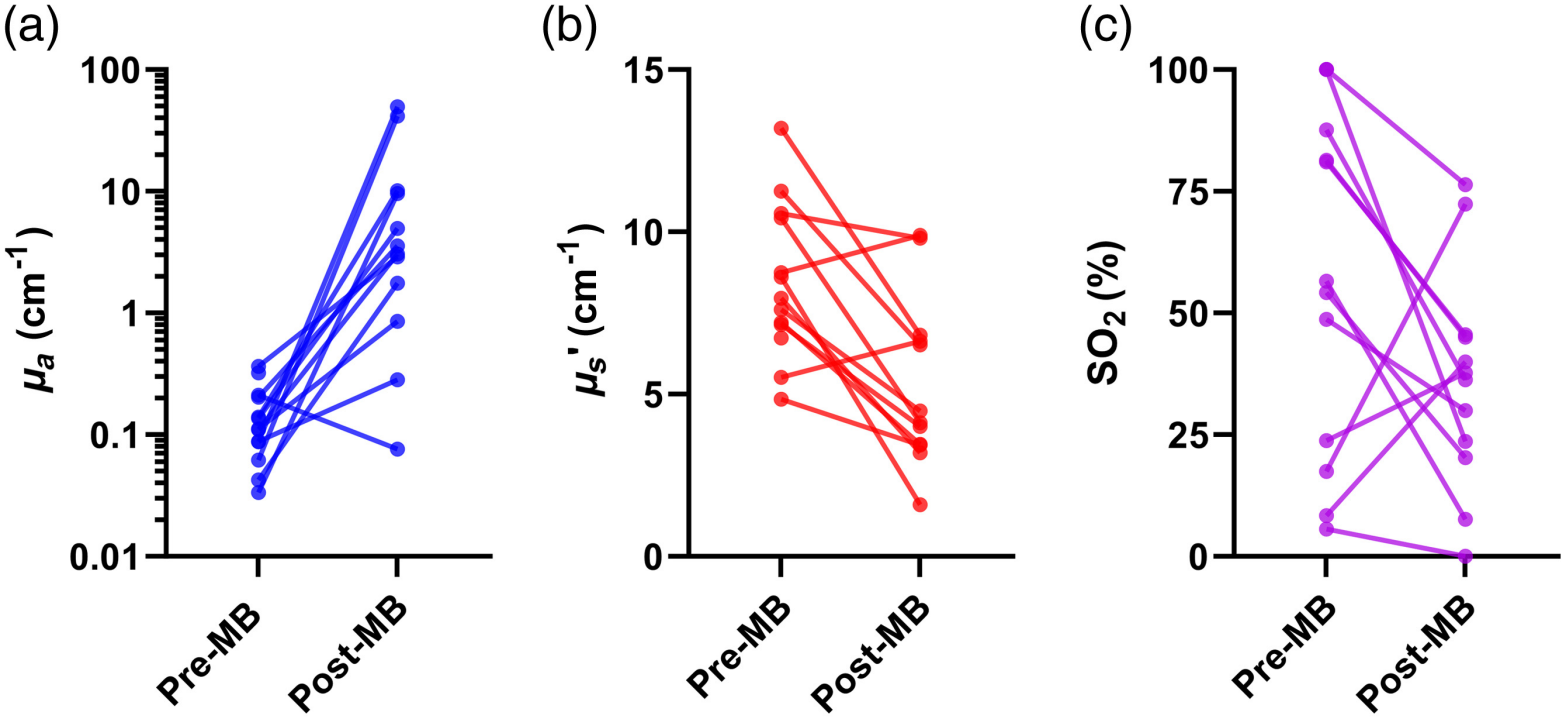
Extracted (a) absorption coefficients ($\mu_{a}$) at 665 nm, (b) reduced scattering coefficients ($\mu_{s}^{\prime }$) at 665 nm, and (c) oxygen saturation (${\mathrm{SO}}_{2}$) for all subjects for pre-MB and post-MB conditions. Note that (a) is on a log scale, and (b) and (c) are on linear scales.

Native, pre-MB abscess wall optical properties at 665 nm across subjects are tabulated in Table 2. Substantial inter-patient variability in native abscess-wall optical properties and oxygen saturation were observed (Fig. 4). Means and standard deviations for absorption coefficient at 665 nm ($\mu_{a,665}$) were $0.15\pm 0.1\;{\mathrm{cm}}^{-1}$ (range: 0.03 to $0.36\;{\mathrm{cm}}^{-1}$), and $8.45\pm 2.37\;{\mathrm{cm}-}^{1}$ (range: 4.8 to $13.2\;{\mathrm{cm}}^{-1}$) for reduced scattering coefficient at 665 nm ($\mu_{s,665}^{\prime }$). Oxygen saturation (${\mathrm{SO}}_{2}$) was found to be 58.83%±35.78% (range: 5.6% to 100%).

**Table 2:** Extracted optical properties at 665 nm and oxygen saturation for each subject, before and after addition of methylene blue (MB). MB concentration ([MB]) is also included for post-MB measurements.

	Pre-MB			Post-MB			
Subject number	$\mu_{a,665}$ (${\mathrm{cm}}^{-1}$)	$\mu_{s,665}^{\prime }$ (${\mathrm{cm}}^{-1}$)	${\mathrm{SO}}_{2}$ (%)	$\mu_{a,665}$ (${\mathrm{cm}}^{-1}$)	$\mu_{s,665}^{\prime }$ (${\mathrm{cm}}^{-1}$)	${\mathrm{SO}}_{2}$ (%)	[MB] (μM)
1^a^	0.32	6.7	100.0	—	—	—	—
2	0.03	10.6	56.5	9.6	9.8	7.6	58.2
3	0.09	5.5	100.0	0.28	6.6	76.4	0.8
4	0.14	7.1	81.1	10.1	4.0	45.5	59.4
5	0.11	8.6	81.4	3.1	1.6	45.1	$1.7\times 10^{-6}$
6	0.09	4.8	87.7	49.4	3.4	36.3	311
7	0.21	8.7	17.4	0.08	9.9	72.4	0
8	0.36	13.2	48.7	2.9	6.8	29.9	8.0
9	0.14	10.4	100.0	5.0	4.1	23.7	13.2
10	0.20	7.2	8.4	3.6	3.5	40.0	17.1
11	0.06	7.6	54.2	41.5	4.5	20.3	227
12	0.11	11.3	5.6	0.86	6.5	0.0001	4.3
13	0.04	8.0	23.8	1.76	3.2	37.7	7.0

a Post-MB data were not of sufficient quality for interpretation for subject 1.

### 3.4 Methylene Blue Uptake and Changes in Extracted Optical Properties

Measured MB concentrations across subjects are tabulated in Table 2. Mean and standard deviation for this was 71.83±108.22  μM (range: 0−311  μM), which reveals substantial inter-patient variation. As expected, there was a significant increase in $\mu_{a}$ at 665 nm for post-MB measurements (p=0.001), due to the presence of MB absorption [Fig. 4(a)]. There was also an apparent decrease in $\mu_{s}^{\prime }$ at 665 nm [p=0.005, Fig. 4(b)]. While there was a decrease in ${\mathrm{SO}}_{2}$ from pre- to post-MB values [median reduction = 28.8%, Fig. 4(c)], this difference was not significant (p=0.11). Additionally, this change in ${\mathrm{SO}}_{2}$ from pre- to post-MB measurement was not significantly correlated with measured MB concentration (ρ=0.49, p=0.11). There was also no significant difference in MB concentration between those subjects graded as having heavy growth on their standard of care abscess aspirate (4+ as reported by the Clinical Microbiology Laboratory) and those with less than heavy growth (4+: 32.2±30.7  μM, <4: 68.1±120.8  μM; p=0.81).

As the fiber optic probe was removed and reinserted between the pre- and post-MB data collection, some of this variability may also represent small differences in the exact placement of the probe face. Due to clinical time constraints, we were unable to perform repeated measurements to assess variability due to probe placement. This will be an important piece of future studies.

### 3.5 Differences based on Abscess Location or Bacteria Present

For the 13 abscesses that were quantified optically, four were located in the pelvis, five were associated with the colon, and four were found in other locations. While sample sizes were small, we performed preliminary analysis to examine regional differences in optical properties. None of the quantities reported in Table 2 were found to be significantly different between these rough location groupings.

Of the bacterial species found in abscesses measured, the most common was *Escherichia coli* (*E. coli*, n=3). Other abscess cavities were found to contain a variety of other species. While abscess containing *E. coli* tended to have higher MB uptake than those with other bacterial species (97.7±114.7  μM versus 14.8±22.7  μM), this difference was not statistically significant (p=0.17). Four of the 13 abscesses measured were found to contain antibiotic-resistant bacterial strains. MB uptake was not different between strains that were resistant or susceptible to antibiotics (p=0.63), indicating that PDT susceptibility in antibiotic-resistant strains would not be limited by MB concentration.

## 4 Discussion and Conclusion

We performed clinical spatially resolved diffuse reflectance spectroscopy of human abscess cavities prior to PDT, representing the first time that the optical properties of human abscesses have been quantified. There was substantial variation in the native abscess wall optical properties prior to MB administration, particularly in absorption at 665 nm and ${\mathrm{SO}}_{2}$. Uptake of MB, as measured by post-MB diffuse reflectance, varied widely between subjects. Infusion of MB appeared to reduce ${\mathrm{SO}}_{2}$ measured at the abscess wall, with minimal effects on tissue scattering. There were no significant differences based on abscess location or bacteria present. These observations motivate future studies focused on the underlying causes of these observed heterogeneities, particularly for MB uptake.

This represents the first study that measured the optical properties of human abscesses. As such, direct comparisons to perfectly analogous results are impossible. However, other investigators have reported optical properties measured within nearby anatomy in the abdomen. Perhaps the most thorough of these is Morales et al., where diffuse reflectance measurements were made with a surface-contact probe throughout the peritoneal cavity.^16^ This included measurements at the surface of multiple organs, the chest wall, and skin. Pre-MB absorption and reduced scattering coefficients determined in this study fall within the ranges reported by these investigators. Wang et al. also measured optical properties within the peritoneal cavity prior to PDT.^33^ Although their results are provided at 630 nm rather than 665 nm, our reported values are similar to those reported for the peritoneum, small bowel, and large bowel. We have also previously measured the optical properties of excised human kidneys,^31^ finding that absorption and reduced scattering at 665 nm ranged from 0.3 to $1.7\;{\mathrm{cm}}^{-1}$ and 12.5 to $39.4\;{\mathrm{cm}}^{-1}$, respectively. These ranges are again similar to what we find in the present study. Abscesses are not routinely surgically excised, as this significantly increases morbidity and mortality.^34^ Capture of biopsy samples is also not possible, as this could rupture the cavity and lead to sepsis. We therefore do not have patient tissue available to perform optical property quantification via other means. Based on these other reports, however, it appears that our measurements are in reasonable concordance with similar abdominal tissue. Future studies will include repeated measurements for the same subject, to evaluate optical property homogeneity and variability in the measurement and fitting process.

Oxygen saturation (${\mathrm{SO}}_{2}$), as determined by the relative concentrations of oxy- and deoxy-hemoglobin, was found to decrease from the pre- to post-MB measurements (55.4%±35.1% versus 36.2%±22.7%) and this decrease was correlated with measured MB concentration (ρ=0.49). However, this change was not statistically significant (p=0.11). This suggests that the infusion of a high MB concentration, even after flushing the cavity with sterile saline, may be related to a reduction in ${\mathrm{SO}}_{2}$. One potential explanation is localized oxidation of hemoglobin to methomoglobin, which cannot bind oxygen,^35^ at high MB concentrations.^36^ As we rely on the balance between oxy- and deoxy-hemoglobin to determine ${\mathrm{SO}}_{2}$, any changes in the ability of hemoglobin to bind oxygen would be incorrectly interpreted as a reduction in the availability of oxygen. Other studies have shown that systemic administration of MB can also result in artificially low ${\mathrm{SO}}_{2}$ readings on standard pulse oximetry.^37^^–^^39^ This could be examined further by including *in situ* measurements with an oxygen-sensitive microelectrode, as we have done previously in phantom studies.^40^ Importantly, we did not observe changes in ${\mathrm{SO}}_{2}$ with standard of care pulse oximetry on the finger, indicating that these changes are localized to the region of MB administration.

**Fig. 5:**
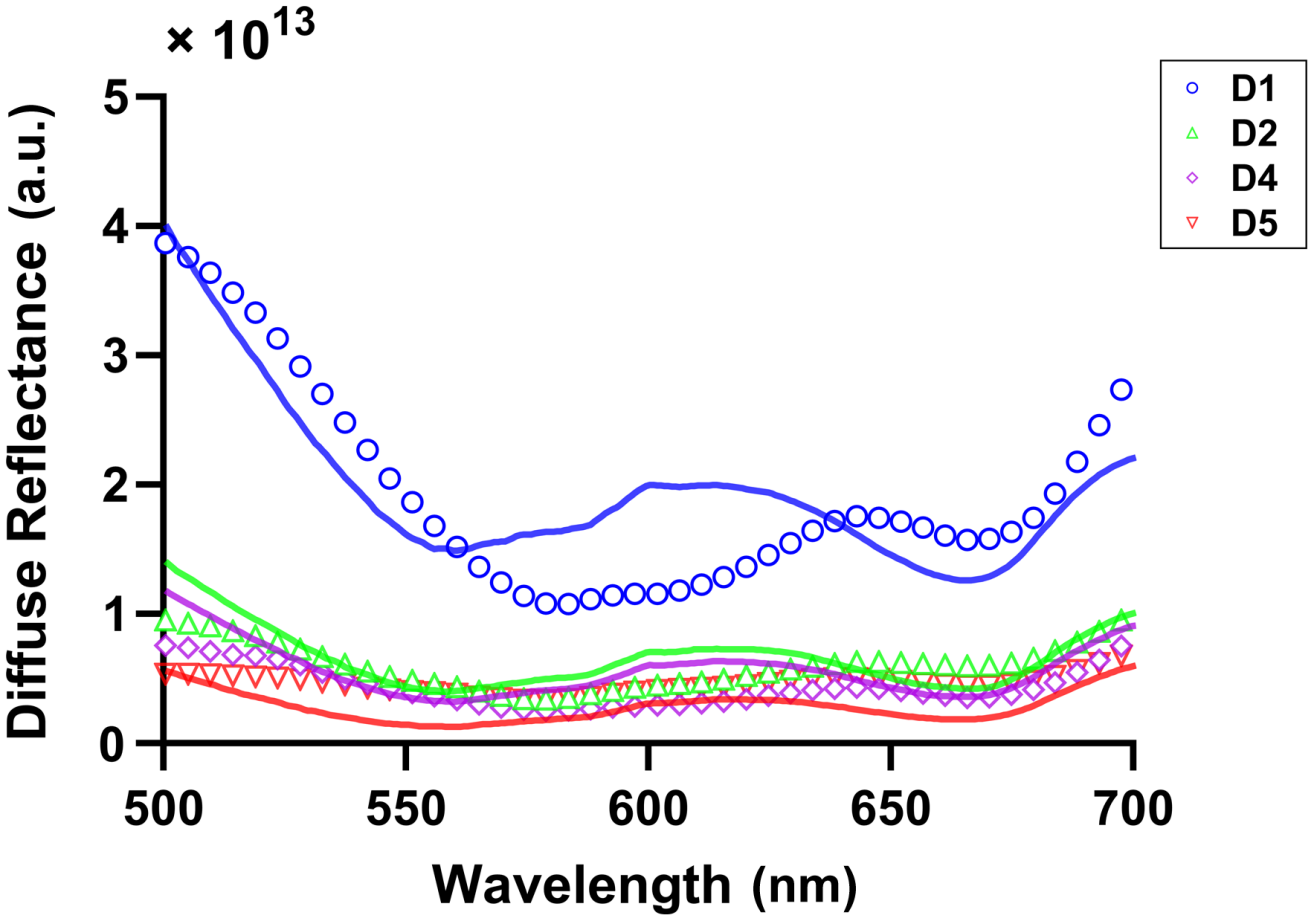
Effect of excluding MB dimer from fitting for the same subject shown in Fig. 2(d). Colors and symbols represent different detector fibers.

As described in Sec. 2.2, we use a multispectral fitting procedure that includes absorption contributions from hemoglobin (oxy- and deoxy-) and MB (monomer and dimer). For the range of MB concentrations we observed here, the inclusion of MB dimer absorption was of particular importance for post-MB measurements. MB forms dimers in aqueous solution, with the dimer fraction correlated with MB concentration.^41^ The absorption of this dimer is shifted from the monomer peak of 664 nm to approximately 600 nm. In addition to more accurate fitting in the spectral region around 600 nm, the inclusion of MB dimer absorption also had a profound effect on the accuracy of ${\mathrm{SO}}_{2}$ determination. An example of this is shown in Fig. 5. Without the MB dimer in the fit the spectral shape is not fit well and an erroneously low ${\mathrm{SO}}_{2}$ value is returned. When the MB dimer is properly included [see Fig. 2(d)], the fit is of much higher quality and the correct value of ${\mathrm{SO}}_{2}$ is found. For the example shown in Fig. 5, the ${\mathrm{SO}}_{2}$ value increased from 0% to 29.9% by inclusion of the dimer. Across all subjects, if fits were performed with the MB dimer absorption included, the determined ${\mathrm{SO}}_{2}$ post-MB was significantly increased (36.2%±22.7% versus 25.9%±27.3%, p=0.027). This highlights the importance of incorporating all relevant absorbers into spectral fitting.

As mentioned previously, MB dimer formation generally occurs at high MB concentration. Taken together with the observation of negative correlation between MB uptake and ${\mathrm{SO}}_{2}$, this motivates reduction of MB concentration in future studies. This is further supported by pre-clinical studies, where MB concentration is typically much lower than the 1  mg/mL concentration used here. For example, Haidaris et al. showed efficacious PDT against multiple bacterial species isolated from patient abscess aspirates at a MB concentration of 300  μg/mL.^7^ In an *in vivo* study in cows, Sellera et al. showed that a MB concentration of 100  μg/mL (0.01%) was efficacious in treating bovine skin infection when combined with red light-emitting diode (LED) illumination.^42^ This 0.01% (100  μg/mL) MB concentration was also shown to be efficacious in controlling *Streptococcus mutans* biofilms.^43^ For clinical studies of MB-PDT, concentrations ranging from 25 to 100  μg/mL have typically been reported,^44^^–^^46^ with some investigators using higher concentrations for topical application.^47^ However, these studies using higher concentrations typically employ much higher fluence rates,^48^ which are not applicable in the case of abscesses.

The wide variability in extracted optical properties, particularly after the addition of MB, provides a strong motivation for the performance of patient-specific treatment planning in future PDT of abscesses. In prior retrospective studies, we have shown the importance of this for improving eligibility for PDT.^25^^,^^26^ Further, the optical properties reported here have been used to generate retrospective treatment plans for the 13 subjects that received both PDT and spectroscopy.^24^ In particular, the effects of abscess wall absorption have a pronounced effect on generated treatment plans. Along with the evidence discussed above, this motivates a reduction in MB concentration in future studies. While prior studies in oncological PDT have employed patient-specific treatment planning,^11^^–^^13^ none of these studies have directly compared patient-specific treatment plans to “fixed dose” plans that do not incorporate patient-specific optical properties. This motivates a direct comparison between a “fixed dose” case and patient-specific treatment planning in upcoming clinical trials.

We acknowledge some limitations in the present study. These findings are from a relatively small (n=13) group of subjects treated with PDT at the time of their abscess drainage, so results could be vulnerable to bias. While a large number of the abscesses measured presented in the pelvis (n=4), many were located in other anatomical locations. There is therefore insufficient sample size to make conclusive statements about regional differences in optical properties. Additionally, extraction of optical properties required the fiber optic probe to make surface contact with the abscess wall. While we used both tactile and optical feedback to verify this, it was apparent in some cases that certain detector fibers were not in contact with the abscess wall. This meant that extraction of optical properties in these cases was based upon a smaller number of detector fibers, which could reduce precision. Further, measurements were only performed at a single location on the abscess wall. These factors motivate the design of alternate probe geometries for future studies, as well as evaluation of the spatial heterogeneity of optical properties and MB uptake. Also, although the saline flushing procedure after MB incubation was identical between subjects, the concentration of MB in extracted saline was not directly measured. This would have provided confirmation that observed differences in apparent MB uptake were not due to the saline flushing process. Future studies will capture the extracted saline after flushing, in order to control for this factor. Finally, while measurements were made before and after the addition of MB, no measurements were made after PDT. This has been done by other groups for PDT of cancer,^16^^,^^49^ to quantify photosensitizer bleaching and PDT-induced changes in optical properties.

## Data Availability

The data that support the findings of this article are not publicly available due to privacy concerns, as research subjects did not consent to use of their data beyond the purposes of this study.
